# Acute Bilateral Lower Extremity Paralysis Secondary to Acute Thrombosis of an Infrarenal Abdominal Aortic Aneurysm

**DOI:** 10.12945/j.aorta.2017.16.045

**Published:** 2018-09-24

**Authors:** T. Joseph Watson, W. Kurtis Childers, Lindsey Haga, John Calaitges

**Affiliations:** 1 Department of General Surgery, Pinnacle Health System, Harrisburg, Pennsylvania, USA; 2 Department of Vascular Surgery, Holy Spirit Hospital System – Geisinger Affiliate, Harrisburg, Pennsylvania, USA

**Keywords:** Thrombosis, Abdominal aortic aneurysm, Paralysis

## Abstract

We present the case of a 64-year-old male who presented to the emergency department with bilateral limb ischemia and paralysis for approximately 1 hour. Computed tomographic angiography demonstrated occlusion of the infrarenal aorta beginning just above the patient’s known abdominal aortic aneurysm (AAA) and extending into both common iliac arteries. He was emergently treated via open repair of the AAA with a Gore-Tex tube graft, bilateral common iliac thrombectomies, and bilateral lower extremity four-compartment fasciotomies. Post-operatively, he had monophasic signals in both posterior tibial arteries, neither of which was present before the operation. During recovery, he developed an ileus but otherwise did not have complications. He was discharged to rehabilitation on post-operative day 15.

## Introduction

Aneurysms are defined as focal dilatations at least 50% larger than the expected normal arterial diameter 1 . A practical working definition of an abdominal aortic aneurysm (AAA) is a transverse diameter of ≥ 3 cm and of a common iliac aneurysm is a transverse diameter > 1.8 cm based on average values for normal individuals. The normal aortic diameter gradually decreases from the thorax (28 mm in men) to the infrarenal location (20 mm in men) 2 . Rather than being termed atherosclerotic, AAAs are more accurately referred to as degenerative or nonspecific in etiology. Degenerative aneurysms account for more than 90% of all infrarenal AAAs 3 .

In contrast to peripheral aneurysms, acute thrombosis of an AAA is a rare sequela 4 . Peripheral aneurysms have an inherent risk of acute thrombosis, which is commonly described for aneurysms of the popliteal artery. An intraluminal thrombus is present in approximately 70–80% of patients with an AAA. This thrombus is usually of no significance to blood flow and may even have some protective effects against the wall stress associated with an AAA 5 . Occlusion of the AAA from an intraluminal thrombus is an extremely rare pathology with an accompanying high morbidity and mortality. This entity was first described in a case report by Schumaker in 1959 and again by Janetta et al. in 1961 4 6 , and the first case series was published by Johnson et al. in 1974 7 . As described by Criado, there are three proposed mechanisms that precipitate acute occlusion of an AAA 8 . The first proposed mechanism stems from occlusive iliac artery disease, leading to aneurysm outflow obstruction as the most common causative factor. The second proposed mechanism occurs after cardioaortic embolization, leading to distal aortic occlusion by a saddle embolus. The third and least common etiology is progression of intrasaccular mural thrombus, which becomes obstructive to blood flow if a sudden change in position occurs, such in a patient fall. Furthermore, in the age of endovascular aortic aneurysm repair (EVAR), one may argue that a fourth potential mechanism may be the clot being provoked by foreign graft material.

Clinical presentations of acute thrombosis of an AAA are often relatively easy to diagnose secondary to acute onset of severe symptoms. In Criado’s review, mottling was described to the level of the iliac crest or umbilicus in 14 out of 26 cases 8 . Also, in Johnson’s et al. review, paraplegia was described at the time of presentation in 10 out of 17 patients 1 .

## Case Presentation

**Figure 1.**
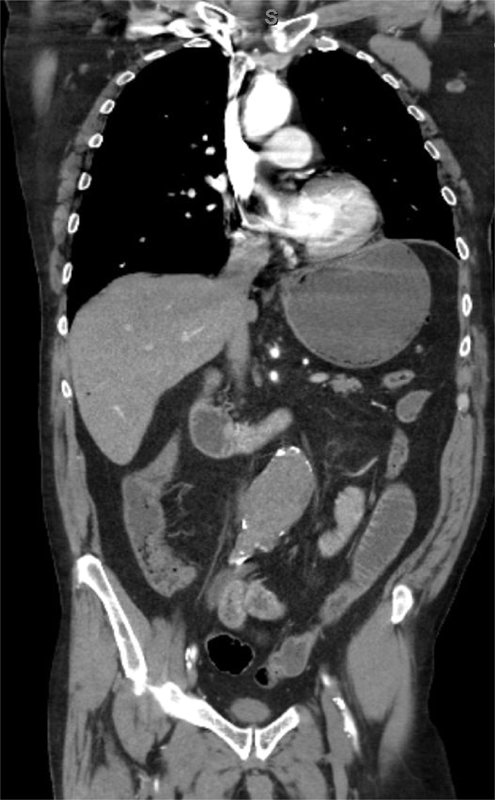
Computed tomography angiography coronal reformat showing thrombosed abdominal aortic aneurysm.

**Figure 2.**
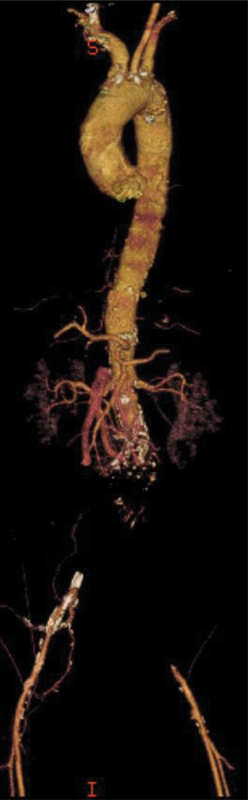
Three-dimensional computed tomography angiography reformat showing acute occlusion of the infrarenal aorta with distal reconstitution in the iliac arteries.

A 64-year-old man presented to the emergency room (ER) complaining of sudden onset bilateral lower extremity pain and numbness that had progressed to paralysis, parasthesias, and stabbing back pain at the time of presentation, which was approximately 1 hour after onset. He did not have a significant past medical history; although, he was a smoker (30 pack-years). His initial vital signs while in the ER were a blood pressure of 139/88, heart rate of 102 bpm, respiratory rate of 20, and oxygen saturation of 94% on 4-L nasal cannula. Physical exam demonstrated mottled bilateral lower extremities with the right being more mottled than the left. Motor strength was diminished in the left (3/5) and absent on the right (0/5). The ER physician was unable to palpate or appreciate a doppler signal bilaterally in the popliteal, posterior tibial, and dorsalis pedis arteries. The patient was alert and oriented but diaphoretic and in obvious distress. Neurological exam was significant for complete sensory loss in the right lower extremity and diminished in the left. Cardiovascular, pulmonary, and abdominal exams were within normal limits. Duplex exam was ordered, but it was unable to be completed secondarily to low flow in the common femoral arteries bilaterally. Velocities ranged from 5.4–6.9 cm/sec and 9.6–11.6 cm/sec on the right and left side, respectively. The patient subsequently underwent a computed tomography angiography (CTA) scan of his chest, abdomen, and pelvis. This revealed a 5-cm infrarenal aortic aneurysm that was acutely thrombosed with extension into the bilateral common iliac arteries ( Figures 1 and 2 ). There was reconstitution in both extremities at the level of the internal and external iliac arteries. Complete blood count and chemistries were drawn, and all were within normal limits.

Consultation with vascular surgery led to a recommendation of emergent operative intervention. The patient gave informed consent and was transferred to a nearby hospital for definitive management. He remained hemodynamically stable during this time; although, the weakness in his left lower extremity progressed to paralysis at the time of transfer.

**Figure 3.**
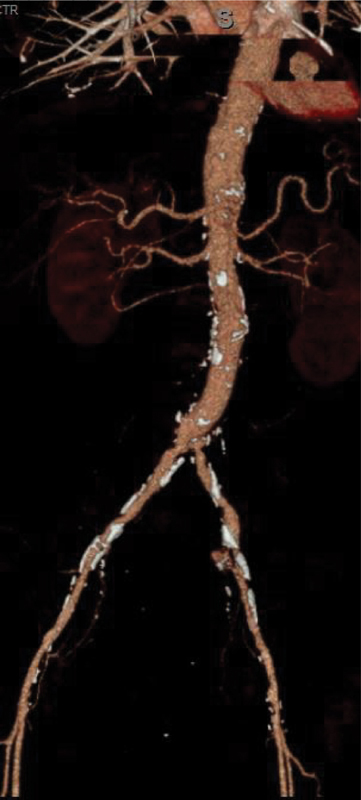
Three-month follow-up computed tomography angiography showing patency.

The patient underwent open repair of his AAA with a Gore-Tex tube graft, bilateral common iliac thrombectomies, left common femoral artery exploration, open thrombectomy with repair, and bilateral lower extremity four-compartment fasciotomies. Upon completion of the surgery, he had monophasic signals in the posterior tibial arteries bilaterally that were not appreciated at the start of the surgery. He remained intubated and was taken to the cardiothoracic intensive care unit in stable condition. On post-operative day 1, he was extubated and was able to move both lower extremities. The posterior tibial signals remained intact. He remained in the cardiothoracic intensive care unit for 2 days before being transferred to the floor. Despite low output from his nasogastric tube, which was removed on post-operative day 3, his recovery was prolonged by a post-operative ileus, necessitating reinsertion of the nasogastric tube as well as parenteral nutrition. The patient continued to work with physical therapy and maintained perfusion and function of his bilateral lower extremities. He was ultimately discharged to an acute rehabilitation facility on post-operative day 15. Upon discharge, he maintained signals bilaterally in the posterior tibial arteries and ambulated with a walker. He has since followed up as an outpatient and is doing well. Three-month CTA confirmed graft patency ( Figure 3 ).

## Discussion

Unlike peripheral aneurysms, acute occlusion of an AAA is a rare entity that carries high morbidity and mortality when diagnosis and treatment is delayed 1 2 4 6 7 8 9 . Mortality rates can reach as high as 46%, which approaches that of aneurysmal rupture 8 . Regardless of etiology, both direct consequences of acute ischemia as well as those associated with revascularization subject the patient to considerable risk 9 . Skeletal muscle injury often occurs within 2 hours of ischemia, and in the presence of paralysis, ultimate limb loss is likely within 6 to 8 hours after onset of acute arterial occlusion 9 10 . Our patient presented with signs of severe lower limb ischemia that began as severe abdominal pain accompanied by paresthesia of the lower extremities. This rapidly progressed to mottling of the lower extremities up to the iliac crest level, and his paresthesia rapidly progressed to complete paralysis of the bilateral lower extremities. The initial response to sudden occlusion of an artery is distal spasm in the main channels and collaterals, which is thought to prevent further thrombosis distal to the occlusion 7 9 . The motor deficit in the lower extremities can be explained in part by anterior spinal cord syndrome secondary to acute occlusion of lumbar arteries 9 11 12 . The motor deficit can also theoretically be attributed to sudden acute ischemia to the lower extremity muscles, ultimately leading to neuronal and muscle ischemia. Therefore, we postulate that the paralysis was multifactorial in nature, from both an anterior spinal cord syndrome as well as prolonged skeletal muscle ischemia. Although the diagnosis of a thrombosed aorta by clinical signs and symptoms is not difficult, the clinician must differentiate thrombosed AAA from a saddle embolus 11 . This is paramount because the therapeutic intervention for a saddle embolus is a transfemoral embolectomy, whereas an abdominal aortic occlusion requires revascularization 10 11 12 . To prevent further clot propagation, it is vital to initiate systemic heparinization after the diagnosis has been established in either situation 1 3 4 6 7 8 10 11 . Emergent surgical repair is mandatory and usually requires thombectomy and replacement of the aneurysm with graft; however, if this is not feasible, an axillobifemoral bypass should be performed 11 13 .

Although thrombosis of an AAA is uncommon, its mortality approaches that of a ruptured AAA. There appears to be no correlation between size and the likelihood of thrombosis, although most reported thromboses have been in smaller aneurysms 12 13 . Once the diagnosis has been established, systemic heparinization is crucial for preventing further propagation of the clot and should be followed by emergent revascularization.
